# Comparison between doppler derived strain and strain rate imaging and cardiac magnetic resonance in assessing right ventricular function late after repaired tetralogy of fallot

**DOI:** 10.1186/1532-429X-13-S1-P216

**Published:** 2011-02-02

**Authors:** Majid Kyavar, Anita Sadeghpour, Shabnam Madadi, Leili Ebrahimi, Zahra Khajali, Zahra Alizadeh, Pedram Golnari

**Affiliations:** 1 Rajaei Cardiovascular Medical and Research Center, Tehran, Iran, Islamic Republic of

## Introduction

Evaluation of right ventricular function is still technically challenging because of the complex anatomy of the RV. CMR has been used as the gold standard for quantitative assessment of RV function, but it is not used routinely in clinical settings since it is expensive and sometimes contraindicated

## Purpose

We sought to evaluate right ventricular (RV) function by Cardiac Magnetic Resonance (CMR) and compare it with Doppler derived strain and strain rate (SR) imaging in patients with repaired tetralogy of fallot( TOF).

## Methods

34 patients (14 women, mean age = 21 ± 4.1 years) late after total correction of tetralogy of Fallot were evaluated. Absolute values of peak systolic strain and strain rate (SR) in the basal, mid, and apical segments of the RV free wall were measured and each segment and mean of three segments were compared with RV ejection fraction measured by CMR.

## Results

RV function measured by CMR was negatively correlated with peak systolic strain of mid RV free wall(P=0.038, Pearson r=-0.357) and also negatively correlated to both mean of strain (P=0.028, Pearson r=-0.376) and SR (P=0.035, Spearman’s rho=-0.362) of basal , mid and apical segments of RV.

## Conclusions

Overall, in the assessment of RV function , echocardiographic measurement of strain and SR and peak systolic strain of mid RV free wall have significant correlation with RV function measured by CMR.

